# Efficacy of anti‐severe acute respiratory syndrome coronavirus 2 mRNA vaccines in adults with severe acquired aplastic anemia with or without allogeneic hematopoietic stem cell transplantation

**DOI:** 10.1002/jha2.570

**Published:** 2022-09-28

**Authors:** Alice Garnier, Amandine Le Bourgeois, Thierry Guillaume, Pierre Peterlin, Maxime Jullien, Marianne Coste‐Burel, Marie C Béné, Patrice Chevallier

**Affiliations:** ^1^ Department of Clinical Hematology CHU de Nantes Nantes France; ^2^ Virology Nantes University Hospital Nantes France; ^3^ Hematology Biology CHU de Nantes Nantes France; ^4^ CNRS, INSERM, CRCI2NA Université de Nantes Nantes France

**Keywords:** allogeneic, aplastic anemia, BNT162b2, COVID‐19, SARS‐CoV‐2 mRNA, third dose, vaccine

Vaccines against severe acute respiratory syndrome coronavirus 2 (SARS‐CoV‐2) have shown remarkable efficacy and thus constitute an important preventive option against coronavirus disease 2019 (COVID‐19) infections, especially in immunocompromised hosts. Several studies have now demonstrated a good response to SARS‐CoV‐2 mRNA vaccines in allogeneic hematopoietic stem cell transplant (Allo‐HSCT) recipients [1, 2, 3]. Aplastic anemia (AA) [4] represents another immunocompromised situation. Surprisingly, the incidence of COVID‐19 infection in this setting appears to be relatively low (2.5%–13%) [5, 6], despite most patients being still on immunotherapy. This could be related to favorable host factors, such as young age, or be secondary to some protective immune dysregulation known to be present in AA [7]. In a recent study of 23 non‐vaccinated AA patients, including seven with a previous Allo‐HSCT, 29% had to be hospitalized after COVID‐19 infection, one died and 65% showed a decline in blood parameters with worsening of cytopenias and increasing need for transfusions.[6] Some data exist also on post‐vaccination results after treatment by horse antithymocyte globulin (hATG) and cyclosporin (CsA) [8, 9] while a few cases of AA have been possibly related to COVID‐19 infection [10, 11] or vaccination [12, 13, 14, 15]. However, as yet, no specific results are available regarding vaccines for AA patients who received an Allo‐HSCT.

This real‐life monocentric observational study aimed at describing the incidence and severity of COVID‐19 infections and humoral responses after anti‐SARS‐CoV‐2 mRNA vaccination (0 to 4, V1, V2, V3, or V4) in a cohort of 27 adults with acquired severe AA (ASAA) who received either an allo‐HSCT (*n* = 13) or hATG+CsA (*n* = 14) between 2012 and 2022 in our Hematology Department. Data were collected in personal file records, during routine follow‐up visits, or using a phone contact. COVID‐19 infection severity was defined by the need for hospitalization and/or subsequent related death. Antibody responses to the SARS‐CoV‐2 spike protein receptor–binding domain were also considered (all tests using Elecsys, Roche, Rotkreuz, Switzerland), the highest value being > 2500 BAU/ml. Based on a threshold of 250 BAU/ml, responses were classified as “weak” or “good”, higher levels having been shown to correlate with the rate of neutralizing antibodies [2]. The study has been approved by the Ethic Review Board of CHU of Nantes and patients gave informed consent.

Patient characteristics are given in Table 1. None of the patients had presented a symptomatic or asymptomatic COVID‐19 infection before V1. Afterward, almost half of the patients (*n* = 13, 48%) were documented with COVID‐19 infection, mostly as a non‐severe form (*n* = 12, 92%, asymptomatic *n* = 3 and mild *n* = 9). Only one severe COVID‐19 infection occurred, required hospitalization in the intensive care unit for 1 month, and issued in full recovery. This severe form occurred also in the only patient who had not been vaccinated. He had been treated with hATG+CsA 18 months earlier and was still receiving CsA. The majority of COVID‐19 infections (*n* = 11/13) occurred between January and July 2022, a period where the Omicron variant was predominant in France. Only two infections had occurred during the Delta period, one in November 2021 and one in December 2021.

**TABLE 1:** Patient characteristics

Patients (*N* = 27)	
Gender: Male/female	14/13
Median age: years old (range)	55 (20–75)
Disease	
Idiopathic/ paroxysmal nocturnal hemoglobinuria	15/12
Previous treatment	
Allotransplant (before the first vaccine)	13 (3)
hATG+CsA (before first vaccine)	14 (3)
Median delay: days(range), between:	
Previous treatment and first vaccine (*n* = 20)	837 (433–3257)
Previous treatment and time of analysis (July 2022)	1098 (48–3702)
Allotransplant and time of analysis (July 2022)	1079 (95–2776)
hATG+CsA and time of analysis (July 2022)	1211 (48–3702)
Ongoing immunosuppressive therapy (CsA)	
Allotransplanted patients	7/13
hATG+CsA patients	12/14
Date of the first vaccine (V1) *n* = 26	January 11–August 18, 2021
Date of second vaccine (V2) *n* = 26	February 1–September 8, 2021
Date of third vaccine (V3) *n* = 23	April 29, 2021–March 15, 2022
Date of fourth vaccine (V4) *n* = 9	November 24, 2021–June 9, 2022
Number of vaccines: 0/2/3/4	1/3/14/9
Type of vaccine (*n* = 84 shots)	
BNT162b2 mRNA (Pfizer‐BioNTech)	80
mRNA 1273 (Moderna)	2 (two patients)
ChAdOx1 nCoV‐19 (Oxford‐AstraZeneca)	2 (same patient)
Preventive treatment by tixagevimab + cilgavimab (Evusheld®)	4

Abbreviations: CsA: cyclosporine A.; hATG: horse anti‐thymoglobulin.

The incidence of infection was similar between allo‐HSCT (8/13) and ATG+CsA (5/14) cases (*p* = 0.26), as well as in patients with idiopathic ASAA (8/12) or paroxysmal nocturnal hemoglobinuria (5/15; *p* = 0.13). No difference either was seen between cases still under immunosuppressive therapy (CsA; 3/8) or not (10/19; *p* = 0.76) or between patients vaccinated before (5/6) or after (8/20) Allo/hATG+CsA (*p* = 0.16). All 3 patients who received only two vaccines contracted the infection while the incidence of COVID‐19 infection was significantly lower in patients with four vaccines (11%) vs those with three (57%; *p* = 0.05).

After a median time of 36 days (range: 20–232) post V2, 75% (*n* = 12/16) of the patients showed a good antibody response, 11 reaching the highest IgG titer, and this proportion increased after V3 and V4. Indeed, at a median time of 70 days (range: 17– 221) after V3, 95% of the patients (*n* = 19/20) had developed a strong antibody response, 17 reaching the highest IgG titer. At a median time of 60 days (range: 2–195) after V4, 100% (*n* = 7/7) of the patients showed a good antibody response, four reaching the highest IgG titer.

Of note, conversely to non‐vaccinated patients [6], no significant worsening of cytopenias was seen after COVID‐19 infection in these patients.

In this cohort, almost half of adults with ASAA presented with COVID‐19 infection with no difference between patients previously allotransplanted and those who had received only hATG+CsA. The incidence was relatively high, but infections occurred mainly during the Omicron wave, confirming the higher contagiousity of this variant over the Delta one. Prolonged exposure to CsA did not appear to affect this incidence and no significant impact on blood counts was observed. Remarkably, while occurring in around one‐third of AA non‐vaccinated patients [6], here less than 10% of the vaccinated patients presented a severe form of COVID‐19 infection, indicating a very good efficiency of the vaccines. This protection increased with the number of shots as the incidence of COVID‐19 was lower and the number of patients reaching good humoral response higher after a second booster. As the latter seems to be more protective, it should be proposed to all ASAA adult patients for whom systemic humoral immune responses appear to be efficient. Of note, such boosts probably also reinforce cellular immunity, the most efficient against viral infections.

## AUTHOR CONTRIBUTIONS

Alice Garnier and Patrice Chevallier designed, performed, coordinated the research, analyzed, performed statistical analyses, interpreted the data, generated the figure, and wrote the manuscript.

Amandine Le Bourgeois, Thierry Guillaume, Pierre Peterlin, and Maxime Jullien recruited patients and commented on the manuscript.

Marianne Coste‐Burel performed serology tests, generated the virologic data, and commented on the manuscript.

Marie C Béné performed statistical analyses and commented on the manuscript.

## ADDITIONAL CONTRIBUTIONS

We acknowledge the following individuals for their assistance with the study, none of whom was compensated for his or her contributions:

The paramedical staff of the Hematology Department and the Virology Department.

## CONFLICT OF INTEREST

The authors declare they have no conflicts of interest.

## FUNDING INFORMATION

The authors received no specific funding for this work.
